# Near-infrared spectroscopy as a high-throughput phenotyping method for fusiform rust resistance in loblolly pine

**DOI:** 10.1016/j.plaphe.2025.100066

**Published:** 2025-06-06

**Authors:** Simone Lim-Hing, Anna O. Conrad, Cristián R. Montes, Kamal J.K. Gandhi, Kitt G. Payn, Trevor D. Walker, Caterina Villari

**Affiliations:** a D.B. Warnell School of Forestry and Natural Resources, University of Georgia, Athens, GA, 30602, USA; b USDA Forest Service, Northern Research Station, Delaware, OH, 43015, USA; c Rayonier Inc., Wildlight, FL, 32097, USA; d Terviva, Fort Pierce, FL, 34951, USA; e Department of Forestry and Environmental Resources, College of Natural Resources, North Carolina State University, Raleigh, NC, 27695, USA

**Keywords:** Classification algorithm, Fusiform rust, Loblolly pine, Phenotyping, Vibrational spectroscopy

## Abstract

Fusiform rust, caused by the pathogen *Cronartium quercuum* (Berk.) Miyabe ex Shirai f. sp. *fusiforme*, is the most important disease of loblolly pine (*Pinus taeda* L.) in the U.S., causing millions of dollars in damage each year. Using resistant genotypes has proven a successful strategy to limit the disease, but resistance selection still relies on visual inspection for symptoms, which can lead to misclassification due to human error and the presence of ‘escaped susceptibles’ (i.e., susceptible individuals with no visible symptoms due to either an extended asymptomatic phase of the disease or the lack of adequate disease pressure to become infected). Here, we propose the use of near-infrared (NIR) spectroscopy and chemometrics to improve the accuracy of how phenotypes are rated. We collected and analyzed phloem and needle spectra from 34 non-related families replicated across eight stands in three states in the southeastern region of the U.S. using a portable, handheld NIR spectrometer. We also used a benchtop Fourier-transformed mid-infrared (FT-IR) spectrometer to analyze phloem phenolic extracts of the same samples, as this phenotyping approach has proved successful in other pathosystems. Our results show a moderate association between the phloem spectra and resistance, and models built with NIR spectra were able to classify extremes (i.e., very resistant or very susceptible) with up to 69 ​% testing accuracy. This study provides a framework for using NIR spectroscopy for phenotyping loblolly pine resistance against pathogens and advocates for using alternative technologies in forestry.

## 1 Introduction

Loblolly pine (*Pinus taeda* L.) is the most widely planted timber species in the southeastern United States, a region touted as being the nation's ‘wood basket’ as it provides nearly 60 ​% of the country's timber [1,2]. A major challenge faced in southeastern pine plantations is fusiform rust disease [caused by the fungal pathogen *Cronartium quercuum* (Berk.) Miyabe ex Shirai f. sp. *Fusiforme* – hereafter *Cqf*], which has been considered the most important disease for southern pines for more than seven decades [3]. Fusiform rust is a macrocyclic and heteroecious rust fungus with a complex disease cycle that alternates between oaks and southern pines. Infection of pines is initiated by wind-dispersed basidiospores, which are produced during the summer on oak leaves and which penetrate the pine hosts through needles or succulent bark tissues [4]. Pine trees are most often infected at young ages (one to five years), and after a latent phase that usually spans six to nine months, the fungus causes the proliferation of woody tissues around the site of infection, resulting in galls that deform the stem and often lead to mortality [4,5]. Even if the trees survive, the galls leave them prone to stem breakage, fire damage, and poor form, substantially lowering their value for timber [3,[6], [7], [8]].

Fungicides can be used to manage the disease in nurseries [9]; however, the main strategy for reducing disease incidence of field trees in high risk areas is through the deployment of families that confer high levels of disease resistance [[10], [11], [12]]. Unlike the majority of forest trees and their diseases, which display quantitative resistance (i.e., linked to multiple genes with usually only a variable partial effect), loblolly pine displays qualitative resistance to fusiform rust, where a few discrete inherited genes confer a high level of resistance [[13], [14], [15]]. In loblolly pine, the *Fr* genes are associated with partial or complete resistance against fusiform rust by means of recognizing the *Cqf* avirulence factor [15,16]. Despite having identified the genetic mechanism for resistance, breeders are still challenged with this pathosystem as commercial loblolly pine families display substantial variation in their level of resistance. The variation stems from management history [17], the allelic frequency of *Fr* genes [18], and from the strains of the pathogen itself [19]. Hence, phenotyping and resistance trials are still necessary for managing fusiform rust [16,20].

The most commonly used selection process for loblolly pine resistance to fusiform rust is through forward selection followed by backwards selection: trees that show promising phenotypes are selected for breeding, and their progeny are grown in field test sites and evaluated for multiple years for the presence of galls; depending on the percentage of galls present, the parent/family is considered either susceptible, and thus discarded, or a candidate for further breeding as a resistant line [21]. While traditional phenotyping using visual assessment of symptoms has resulted in tremendous gains in rust resistance [5], it is prone to human error and can be subjective [22,23]. Symptoms can be inconspicuous: for example, galls at the base of the tree are oftentimes obscured by leaf litter, and smaller galls on branches may be hidden behind dense foliage. Less-pronounced symptoms, such as minor swelling or the absence of a gall but the presence of branch proliferation, can cause uncertainty for the field crew. These human errors frequently complicate selection plans, and around 10 ​% of first-round selection candidates are later disqualified due to the discovery of symptoms that were overlooked during phenotyping [24]. Moreover, fusiform rust has a long latent phase in which infected tissues do not show any symptoms [4], which further complicates visual assessment. Development of symptoms also depends on the local disease pressure, which varies locally throughout the southeastern U.S. and over time [10,25]. These factors, along with the already high variability of resistance, can lead to ‘escaped susceptibles,’ or susceptible individuals or families that are incorrectly classified as resistant because they did not present symptoms during their rearing at test sites [26]. Using a phenotyping method that does not rely on the observation of symptoms, in tandem with the traditional screening, would provide an additional layer of confidence in the process, greatly improving the accuracy and efficacy of selection.

As technology advances and becomes more affordable, forest scientists are exploring new ways to improve the management of diseases, including the selection of better-performing trees. One such advance is the use of vibrational spectroscopy as a phenotyping tool. Vibrational spectroscopy-based techniques, such as near-infrared (NIR) or Fourier-transformed mid-infrared (FT-IR) spectroscopy, provide the user with a spectrum that is based on the vibrational patterns of the chemical constituents in a given sample [27]. Vibrational spectroscopy tools range from portable, hand-held devices that can be used in-field for rapid data collection to extremely sensitive benchtop equipment that can detect minute differences in samples [28].

Vibrational spectroscopy has been successfully used as a tool in fields such as food science [29], medical science, such as for cancer diagnostics [30], and toxicology [31]. In biological plant applications, vibrational spectroscopy-based phenotyping can be a powerful non-destructive alternative to genotyping in which spectral data combined with multivariate statistics and machine learning (i.e., chemometrics) can be used to classify samples into groups depending on patterns associated with the sample's chemistry [32]. Successful examples include a range of traits such as drought-tolerance in wheat (*Triticum aestivum* L.) [33], protein quality in pulse crops [34], and cell wall composition in *Petunia hybrida* transgenic plants [35]. Vibrational spectroscopy has also been employed for plant disease diagnostics, being particularly useful for identifying pre-symptomatic plants based on the shift of their chemical composition [36,37]. Recent examples for forest tree diseases include the identification of Norway spruce [*Picea abies* (L.) H. Karst] trees infected with Heterobasidion root disease [caused by *Heterobasidion* annosum (Fr.) Bref.] using FT-IR spectra of phenolic extracts of the xylem and needles [38]*,* and the identification of American beech (*Fagus grandifolia* Ehrh.) leaves infected with beech leaf disease [caused by *Litylenchus crenatae* ssp. *mccannii* Handoo et al.] using foliar spectra collected with a handheld NIR scanner [39]. The use of vibrational spectroscopy with respect to predicting the resistance or susceptibility of naïve (i.e., uninfected) individuals to a given pathogen is more nascent, but the technology offers great promise, as shown by the successful use of FT-IR spectra of phenolic extracts for the classification of resistance against sudden oak death (caused by *Phytophthora ramorum* Werres, de Cock & Man in't Veld) in coast live oak (*Quercus agrifolia* Née) [40], Diplodia tip blight [caused by *Diplodia sapinea* (Fr.) Fuckel (Syn: *Sphaeropsis sapinea* (Fr.) Dyko & Sutton)] in Austrian pine (*Pinus nigra* J.F. Arnold) [41], and ash dieback [caused by *Hymenoscyphus fraxineus* (T. Kowalski) Baral, Queloz & Hosoya] in European ash (*Fraxinus excelsior* L.) [42]. In all these studies, multivariate statistics and machine learning approaches were able to identify a relationship between the chemical constituents reflected in the spectra of non-infected individuals and their resistance phenotype, allowing for the correct prediction of resistance or susceptibility of naïve individuals.

The goal of this study was to assess whether vibrational spectroscopy can be used to supplement the selection process by improving the accuracy of classifying resistance of loblolly pine to fusiform rust. We took advantage of a previously-phenotyped progeny test population, where asymptomatic trees could be assigned as genetically resistant or susceptible based on the mean performance of their relatives (i.e., breeding values). We also utilized two different analytical approaches that have been shown to have promise in other forest systems [32]. We tested the use of a handheld NIR that can be deployed directly in the field, on unprocessed plant tissues [36]. Handheld devices offer a conveniently portable solution that would be ideal for field crews to use, but portability often forgoes the sensitivity and accuracy obtained with benchtop devices. We also tested the use of a benchtop FT-IR with phenolic extracts, which requires a laborious sample preparation but has already been shown to achieve good phenotyping results in other systems (e.g., Ref. [42]).

## 2 Materials and methods

### 2.1 Experimental design and estimation of genetic resistance for asymptomatic trees

**Fig. 1:**
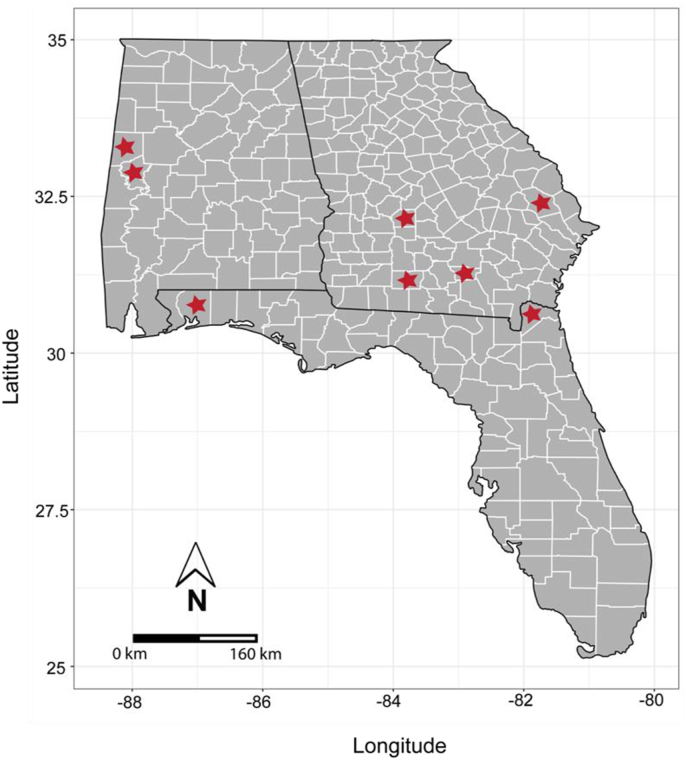
Map of the counties (red stars) in Alabama, Florida, and Georgia where the eight sampling sites are located in the southeastern US. Phloem and needles from 5-yr old *Pinus taeda* families with known resistance levels to fusiform rust (caused by *Cronartium quercuum* f. sp. *fusiforme*) were both scanned by a handheld near-infrared (NIR) device and collected to be processed on a benchtop Fourier-transformed infrared (FT-IR) equipment. Resistance was based on breeding values across progeny tests.

We sampled 5-year-old loblolly pine trees at eight progeny test sites across three states in the southeastern United States (Fig. 1). The trees were part of progeny trials for the 4th-Cycle Coastal breeding population of the North Carolina State University Cooperative Tree Improvement Program (TIP) [43] and were planted in the winter of 2016–2017. The trials included a total of 229 families, with each test site hosting up to 160 families and any two pairs of sites sharing at least 52 common families. There were six to 12 replicates (trees) per family per site. Using traditional visual phenotyping, trees were evaluated at age four years for the presence or absence of fusiform rust symptoms on the main stem or branches. These data, along with assessments from other progeny tests in the cycle, were used to estimate the additive genetic effect (breeding value) for the probability of infection. The genetic effect of an individual reflects the mean performance of its relatives proportional to their coancestry. The genetic effect (probability of infection) was predicted for each tree using a generalized linear mixed model with a logistic link function [44]. The model includes a fixed effect for the intercept, a random effect for the test site, with an independent identically normal distribution with mean zero, and a random effect for each tree, with a normal distribution of variance-covariance proportional to the additive numerator relationship matrix among trees and mean zero [45].

Once genetic resistance was estimated for each tree, we developed a list of trees to sample by removing any trees that had symptoms of rust at the age four assessment. We wanted to sample only non-infected trees to obtain signatures associated with their constitutive chemical composition, rather than any change induced by disease, which is a critical step for the use of vibrational spectroscopy as a resistance phenotyping tool [42]. Next, we identified families having trees with the most and least genetic resistance, and all related families were removed from the target sample list. The process was repeated until there was a list of trees coming from a set of 20 resistant families that were unrelated. The same process was then used to develop a list of the 20 most susceptible and unrelated families. We further removed families that shared a parent across the two groups, thereby selecting a total of 34 unrelated families (20 resistant, 14 susceptible). Not every family appeared at every test site, and each family was represented at a minimum of two to a maximum of eight sites, with the plurality of the families being represented at least at four of the eight test sites. The list of trees in each family was further trimmed so that there would be up to two sample trees for each family within a test site, but trees that showed symptoms of rust or other damage at the time of collection were discarded, resulting in a total of 275 sampled trees (Table 1). Detailed information on the location and number of trees selected for each family is in Table S1.

**Table 1:** Summarized information on the *Pinus taeda* families sampled in the study. Detailed information about the representation of families at each site is reported in Table S1.

State	County	Date of sampling	Number of families (number of replicates per family is given in parentheses)	
			Resistant	Susceptible
Georgia	Colquitt	February 3, 2022	11(2), 2(1)	7(2), 2(1)
Alabama	Greene	February 7, 2022	8(2), 2(1)	5(2)
Alabama	Pickens	February 8, 2022	9(2), 3(1)	8(2), 2(1)
Georgia	Dooly	February 10, 2022	10(2)	5(2), 1(1)
Georgia	Atkinson	February 17, 2022	9(2), 1(1)	5(2), 1(1)
Florida	Nassau	February 18, 2022	11(2), 1(1)	8(2), 1(1)
Georgia	Bulloch	February 25, 2022	9(2), 3(1)	5(2), 4(1)
Florida	Santa Rosa	February 28, 2022	10(2)	5(2), 1(1)

Total number of families			20	14
Total number of trees			167	108

### 2.2 NIR scanning in the field and tissue harvesting

Samples were scanned and collected between 2–28 February 2022. The diffuse reflectance of samples was collected at ambient temperature using a NeoSpectra NIR scanner (Si-Ware Systems, Menlo Park, California, United States) with a scan time of 5 ​s at 3-s intervals, a spectral range of 3920–7380 ​cm^−1^ (2550-1355 ​nm), and a resolution of 16 ​nm as measured at 1550 ​nm. Five technical replicate spectral readings per sample were automatically captured by the device. Before each sample, the device was blanked by scanning a white reference (provided by the device's manufacturer) for 5 ​s, while after each sample, the device's sensor was gently cleaned with 95 ​% isopropyl alcohol (Supleco Inc., Bellefonte, Pennsylvania, USA).

Both twig and phloem needle tissues were scanned since either tissue is easy to collect without excessively damaging the trees, while at the same time is important in the disease development process, and could hence potentially be used for resistance phenotyping applications. We started by excising the tip of a lateral branch at mid-canopy height and with fully-expanded needles using a pole pruner. For scanning the needles, we removed fully-expanded fascicle bundles, bunched them together, and laid them flat on the sensor of the scanner. For scanning the phloem, we removed all needles on the branch, shaved the phloem closest to the tip of the branch using a surgical blade, and laid the tissue flat (phloem side down) to cover the sensor (Fig. S1).

The rest of the needles and phloem shaving from the same branch were bagged individually in labeled paper coin envelopes and immediately frozen and stored in dry ice until they were transported to the laboratory, where they were stored at −80 ​°C until further processing. At one site (Atkinson, Georgia), we could not acquire dry ice for the collection and shipping, hence we did not collect tissue for further laboratory analyses.

### 2.3 Phenolic extraction

Preliminary results from the NIR classification suggested that the phloem's chemical signature is a better indicator of resistance and therefore we processed only the phloem tissues for the FT-IR analysis, which required samples to be in a liquid form (as opposed to the unprocessed tissues analyzed with the NIR scanner). We used phenolic extracts for the analyses because phenolics are known to have a strong association with plant defense mechanisms against biotic attackers [46], and previous studies using FT-IR for phenotyping resistance in trees used spectra collected from phenolic extracts [[40], [41], [42]]. We extracted phenolic compounds following the protocol described in López-Goldar et al. [47]: first, we ground phloem tissues into a fine powder in liquid nitrogen and aliquoted 200±1 ​mg of each sample into 2 ​mL microcentrifuge tubes. We then added 700 ​mL of HPLC-grade methanol (ThermoFisher Scientific, Walthman, Massachusetts, USA), vortexed the solution for approximately 10 ​s, and stored it overnight at 4 ​°C. The next day, we centrifuged the samples at 16,000 RCF for 8 ​min using an Eppendorf Centrifuge 5424 (Eppendorf AG, Hamburg, Germany), transferred the supernatant to a fresh microcentrifuge tube, and repeated the extraction steps from the first day. The leftover pellet after the second day of extraction was discarded. We increased sample concentration 10-fold by drying down 500 ​μL aliquots of samples in an Eppendorf Vacufuge™ (Eppendorf AG) and resuspending them in 50 ​μL of HPLC-grade methanol. Resuspended samples were stored at −20 ​°C in 1.5 ​mL microcentrifuge tubes sealed with Parafilm (ThermoFisher Scientific) until use.

### 2.4 FT-IR analysis

We performed FT-IR analysis of phenolic extracts from phloem tissue on a Nicolet iS50 Spectrometer equipped with an iS50 attenuated total reflectance accessory (ATR) and a Diamond DLaTGS crystal (ThermoFisher Scientific) using the OMNIC software (ThermoFisher Scientific). We placed 1 ​μL of phenolic extract on the diamond crystal and left it to air dry for about 10 ​s before scanning the sample. The FT-IR captured the spectra in the mid-infrared region, with a wavenumber range of 4000-700 ​cm^−1^ (2500–14285 ​nm), co-adding 64 scans for each sample with a resolution of 4 ​cm^−1^ and an aperture of 7, as per Villari et al. [42]. We collected two technical replicates per sample and before each scan, we gently cleaned the diamond crystal using ethanol. We periodically recalibrated the spectrometer by scanning a blank background every three samples.

### 2.5 Data processing and statistical analyses

All data were analyzed using R version 4.2.2 [48]. Before any processing, the five NIR and two FT-IR technical replicate readings were averaged over each sample. To have a preliminary overview of the spectral data, we analyzed both phloem and needles spectra via non-metric multidimensional scaling (NMDS), using the ‘vegan’ package [49]. A non-parametric permutation MANOVA (PERMANOVA) was used on the NMDS distance matrix to test if there were site effects and/or an interaction between site and resistance class.

Data were analyzed using a modified chemometric pipeline previously developed by Conrad et al. [36] for identifying rice (*Oryza sativa* L.) plants infected by sheath blight (caused by *Rhizoctonia solani* J.G. Kühn) using NIR spectroscopy, which used the precursor of the device used in this study. Firstly, outliers were identified and removed using the ‘fda’ and ‘fda.usc’ packages [50,51]. Outliers were identified based on the assumption that, for each sample, the depth of the spectral curve and its outlyingness are inversely related [50]. Next, all spectra were second-derivative transformed using the ‘mdatools’ package [52] with the following parameters as per Conrad et al. [36]: width of filter window ​= ​15, porder ​= ​2, dorder ​= ​2.

We employed two model workflows: 1) support vector machine classification algorithm as implemented in the ‘e1071’ package [53] using variables selected by random forest as implemented in the ‘VSURF’ package [54]; 2) a sparse partial least squares discriminatory analysis (sPLS-DA) as implemented in the ‘mixOmics’ package [55]. Both approaches use variable selection to identify important predictors for classification and cross validation to mitigate overfitting. For the analysis of NIR spectra, we included the whole spectral range captured. For the analysis of the FT-IR spectra, we only included the range of 2430–3540 ​cm^−1^, corresponding to the so called fingerprinting range, which is where individual bonds associated with unique molecules can be characterized and where discriminating power is often the highest [56].

For each classification model, the data were randomly split into training (70 ​% of the data) and testing (30 ​% of the data) sets using the ‘caret’ package [57] to create balanced datasets. In preliminary analyses, the model accuracies varied significantly depending on the way data was randomly split (i.e., setting seed in data partitioning). We thus opted for a bootstrapping approach, performing 100 iterations of each model, each with different data partitions, to minimize the sampling bias and provide a more robust and realistic estimate of model performance. The performance was then evaluated based on the average accuracies and standard errors of the training model (model fit) and of the testing model (predictive accuracy), as well as the average sensitivities (in this case, rate of correctly classifying resistance) and specificities (in this case, rate of correctly classifying susceptibility) of the testing models. For each variable selection model, we identified the top five most frequently occurring wavenumbers across the 100 iterations by ordering index values in a frequency table (Tables 2 and 3). It is important to note that while these individual bands were used to increase model performance by reducing multicollinearity and model overfitting, individual bands cannot necessarily be linked to individual chemical compounds. The goal of this approach was to identify broad-scale patterns associated with disease resistance in the spectra rather than identify individual physiological mechanisms of resistance. As such, we can only speculate on the relationship between a spectral region or band, a chemical compound, and their role in resistance against pathogens.

**Table 2:** Wavenumbers (cm^−1^) collected with the field-based NIR scanner and selected by random forest or sparse partial least squares discriminatory analysis (sPLS-DA) that were used in support vector machine (SVM) and sPLS-DA classification models, respectively. Wavenumbers that reoccur more than once per tissue type are indicated in bold font.

Tissue	Variable selection	Data used	Wavenumber (cm^−1^)
Phloem	Random forest	30 most resistant and 30 most susceptible trees	5787, **5800**, **5814**, **5827**, **5841**
		40 most resistant and 40 most susceptible trees	5664, **5678**, **5800**, **5827**, **6222**
		All breeding values	4044, **5678**, 6154, 6344, 6767
	sPLS-DA	30 most resistant and 30 most susceptible trees	**5814**, 5827, **5841**
		40 most resistant and 40 most susceptible trees	**6222**
		All breeding values	4084, 4425, 5527, 5542, 6685
Needles	Random forest	30 most resistant and 30 most susceptible trees	**5297**, 5705, **5746**, 6004, 6290
		40 most resistant and 40 most susceptible trees	4262, **5297**, 5704, 5719,6440
		All breeding values	4412, 5745, 6004, 6658, 6671
	sPLS-DA	30 most resistant and 30 most susceptible trees	4602, 4970, 4983, 4997, 5759
		40 most resistant and 40 most susceptible trees	4656, 4684, 5528, 5732, **5746**
		All breeding values	6455

**Table 3:** Wavenumbers (cm^−1^) collected with the laboratory-based FT-IR and selected by random forest or sparse partial least squares discriminatory analysis (sPLS-DA) that were used in support vector machine (SVM) and sPLS-DA classification models, respectively. Wavenumbers that reoccur more than once in each tissue type are indicated in bold font.

Variable selection	Data used	Wavenumber (cm^−1^)
Random forest	30 most resistant and 30 most susceptible trees	2603, **2626**, **2719**, 3485, 3489
	40 most resistant and 40 most susceptible trees	2436, **2626**, **2719**, 2986, 3016
	All breeding values	2912, 2913, 2914, 3016, 3218
sPLS-DA	30 most resistant and 30 most susceptible trees	**2437**, 2439, 2440, 2441, 2486
	40 most resistant and 40 most susceptible trees	**2437**, **2491**, 2496, 2497, 2498
	All breeding values	2447, **2491**, 2492, 2494, 2495

To test the models’ capacity of classifying resistance, the data was partitioned by differing degrees of conservatism. The first dataset included only the 30 most resistant and 30 most susceptible trees, based on their breeding value. Excluding intermediate phenotypes when building phenotyping models is a common practice, as models are expected to perform best when built on well-defined groups [32,40,42]. To test how well the models performed, a higher number of intermediates were incorporated. In the second dataset, the number of samples was increased to include the 40 most resistant and 40 most susceptible trees based on their breeding value. Finally, in the third dataset, all samples were used, randomly selecting an equal number of trees per resistance class to balance the analysis.

## 3 Results

### 3.1 NIR phenotyping

A total of 275 phloem and needle tissue samples were collected from eight different progeny test sites across three states in the southeastern US (Alabama, Florida, and Georgia). The NMDS of all NIR spectra showed that there was strong grouping by site, but not by resistance class for both tissue types (Fig. S2 and S3). A PERMANOVA run on the distance matrix validated the visual observations: for phloem tissue, resistance class (R^2^ ​= ​0.008, p ​= ​0.613) and the interaction between resistance class and site (R^2^ ​= ​0.016, p ​= ​0.422) were not significantly grouped, but site was significant (R^2^ ​= ​0.381, p ​< ​0.001). Analysis of the distance matrix for needle tissue yielded similar results, where resistance class (R^2^ ​= ​0.004, p ​= ​0.194) and the interaction between resistance class and site (R^2^ ​= ​0.024, p ​= ​0.168) were not significantly grouped, but the site was again significant (R^2^ ​= ​0.373, p ​< ​0.001).

**Fig. 2:**
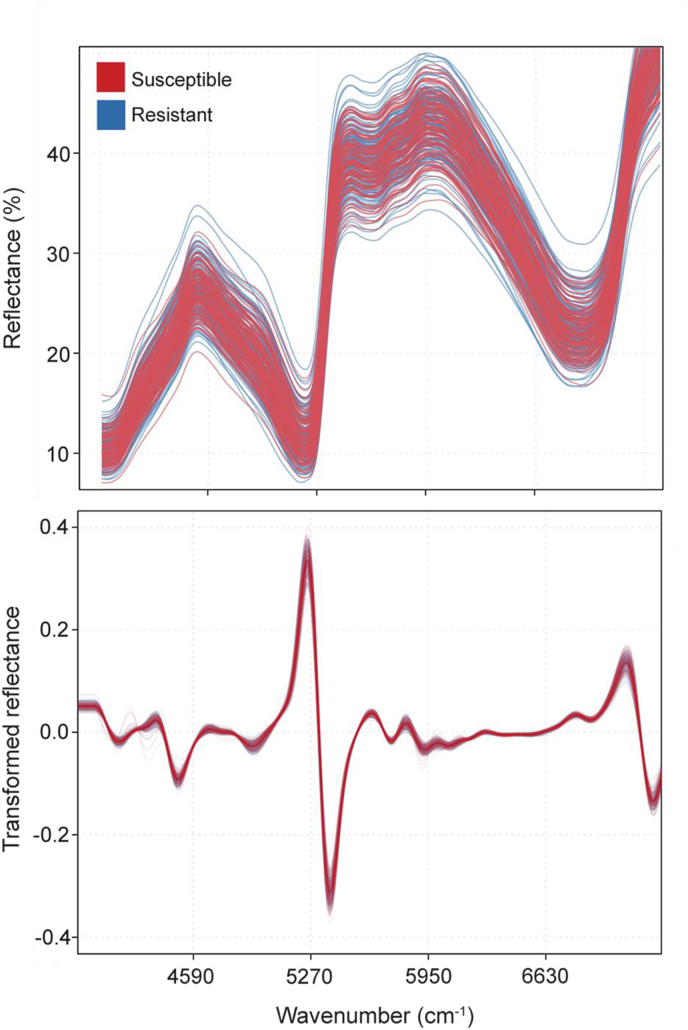
Raw and second-derivative transformed NIR spectra of phloem tissue of 5-yr old *Pinus taeda* seedlings representing families that are either susceptible or resistant to *Cronartium quercuum* f. sp. *fusiforme*, the causal agent of fusiform rust. Resistance was assigned based on breeding values across progeny tests. Spectra were collected with a handled NIR scanner.

**Fig. 3:**
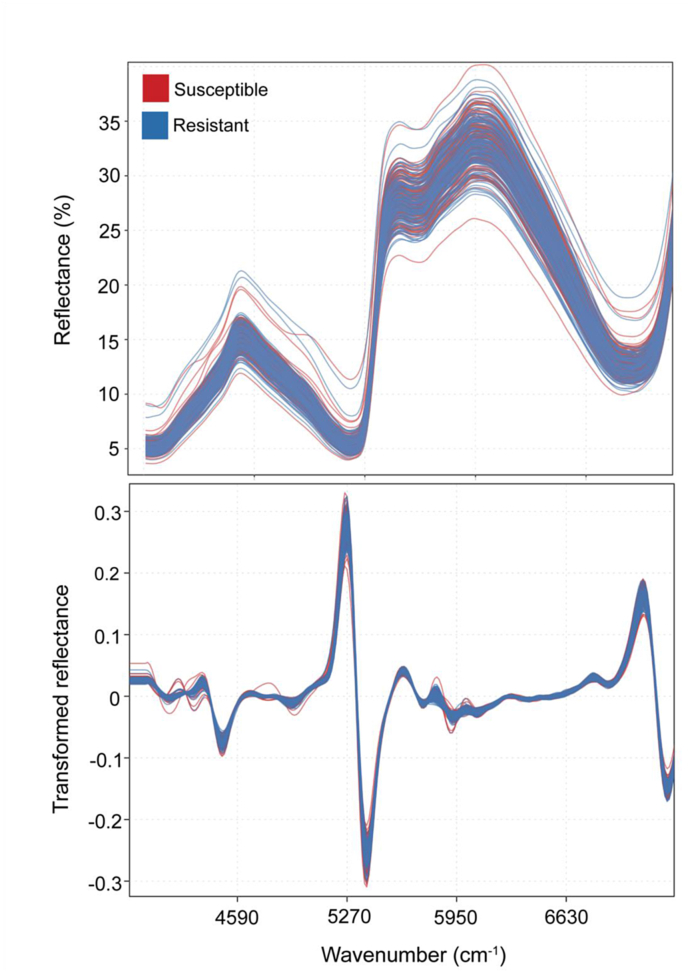
Raw and second-derivative transformed NIR spectra of needle tissue of 5-yr old *Pinus taeda* seedlings representing families that are either susceptible or resistant to *Cronartium quercuum* f. sp. *fusiforme*, the causal agent of fusiform rust. Resistance was assigned based on breeding values across progeny tests. Spectra were collected with a handled NIR scanner.

Seven phloem and nine needle samples were identified by the chemometric pipeline as outliers, resulting in 268 and 266 samples for phloem and needle tissue, respectively. The average raw and second-derivative transformed spectra did not display noticeable differences between resistant and susceptible spectra for both tissue types (Figs. 2 and 3).

The accuracy, sensitivity, and specificity of the supervised classification models were dependent on the variables being used for each model, tissue type, and how the dataset was partitioned. The first approach employed random forest-selected variables paired with SVM as a classification mode. When using this approach, models built with phloem tissue consistently performed better than those using needle tissue, and the accuracy of the models decreased with the decrease of the conservatism in assigning resistance: the model with the highest accuracy was the one built using the 30 most resistant and 30 most susceptible trees, which had a training accuracy of 81.5 ​% and a testing accuracy of 68.1 ​% (Table 4, Set 1). The accuracy of the predictive model decreased when increasing the number of samples in the dataset to include the 40 most resistant and 40 most susceptible trees. Using this expanded data subset, the training accuracy was 70.1 ​% and the testing accuracy was 61.7 ​% (Table 4, Set 2). Overall model accuracies further decreased when including intermediate breeding values, with a training accuracy of 95.0 ​% and a testing accuracy of 55.7 ​% (Table 4, Set 3). The sensitivity and specificity of the different model forms followed a similar pattern, generally decreasing as more intermediate samples were included (Table S2).

**Table 4:** Parameters and average accuracies of the three support vector machine (SVM) models with random forest-selected variables built with field-based NIR spectra and used to predict the resistance of 5-yr old *Pinus taeda* seedlings representing families that are either susceptible or resistant to *Cronartium quercuum* f. sp. *fusiforme*. Training and testing groups were portioned 70 ​% and 30 ​%, respectively, with equal resistance class representation. Model accuracies were averaged across 100 iterations of the data splitting. The best model is indicated in bold font.

Set	Data used	N	Group	Average accuracy ±SE	
				Phloem	Needles
1	30 most resistant and 30 most susceptible trees	60	**Training**	**0.815 ​± ​0.004**	0.940 ​± ​0.003
			**Testing**	**0.681 ​± ​0.009**	0.616 ​± ​0.009
2	40 most resistant and 40 most susceptible trees	80	Training	0.701 ​± ​0.005	0.792 ​± ​0.004
			Testing	0.617 ​± ​0.008	0.604 ​± ​0.009
3	All breeding values	216 (phloem)	Training	0.950 ​± ​0.001	0.894 ​± ​0.002
		210 (needles)	Testing	0.557 ​± ​0.005	0.443 ​± ​0.006

Results with the sPLS-DA supervised classification approach mirrored those obtained with the SVMs, with models built with phloem tissue performing better than those built with needles, and with higher accuracy in models built with conservative data partitioning. When using the 30 most resistant and 30 most susceptible trees, the best predicting model had a training accuracy of 79.5 ​% and a testing accuracy of 68.7 ​% (Table 5, Set 1). For the 40 most resistant and 40 most susceptible trees, the best predicting model had a training accuracy of 70.3 ​% and a testing accuracy of 52.9 ​% (Table 5, Set 2). Model accuracies further decreased when using intermediate breeding values, having a training accuracy of 59.8 ​% and a testing accuracy of 47.3 ​% (Table 5, Set 3). Even for the sPLS-DA supervised classification approach, the sensitivity and specificity of the different model forms mirrored the pattern of accuracies (Table S2). Of the bands identified as being important for classification, either by random forest or sPLS-DA, many were recurring across different models, especially for those built with phloem spectra (Table 2). The most recurring spectra were 5678, 5800, 5814, 5827, 5841, and 6222 ​cm^−1^.

**Table 5:** Parameters and average accuracies of the sparse partial least squares discriminatory analysis (sPLS-DA) built with field-based NIR spectra and used to predict the resistance of 5-yr old *Pinus taeda* seedlings representing families that are either susceptible or resistant to *Cronartium quercuum* f. sp. *fusiforme*. Training and testing groups were portioned 70 ​% and 30 ​%, respectively, with equal resistance class representation. Model accuracies were averaged across 100 iterations of the data splitting. The best model is indicated in bold font.

Set	Data used	N	Group	Average accuracy ±SE	
				Phloem	Needles
1	30 most resistant and 30 most susceptible trees	60	**Training**	**0.795 ​± ​0.004**	0.761 ​± ​0.005
			**Testing**	**0.687 ​± ​0.008**	0.499 ​± ​0.010
2	40 most resistant and 40 most susceptible trees	80	Training	0.703 ​± ​0.003	0.718 ​± ​0.004
			Testing	0.529 ​± ​0.009	0.459 ​± ​0.008
3	All breeding values	216 (phloem)	Training	0.598 ​± ​0.002	0.588 ​± ​0.002
		210 (needles)	Testing	0.473 ​± ​0.006	0.470 ​± ​0.006

### 3.2 FT-IR phenotyping

**Fig. 4:**
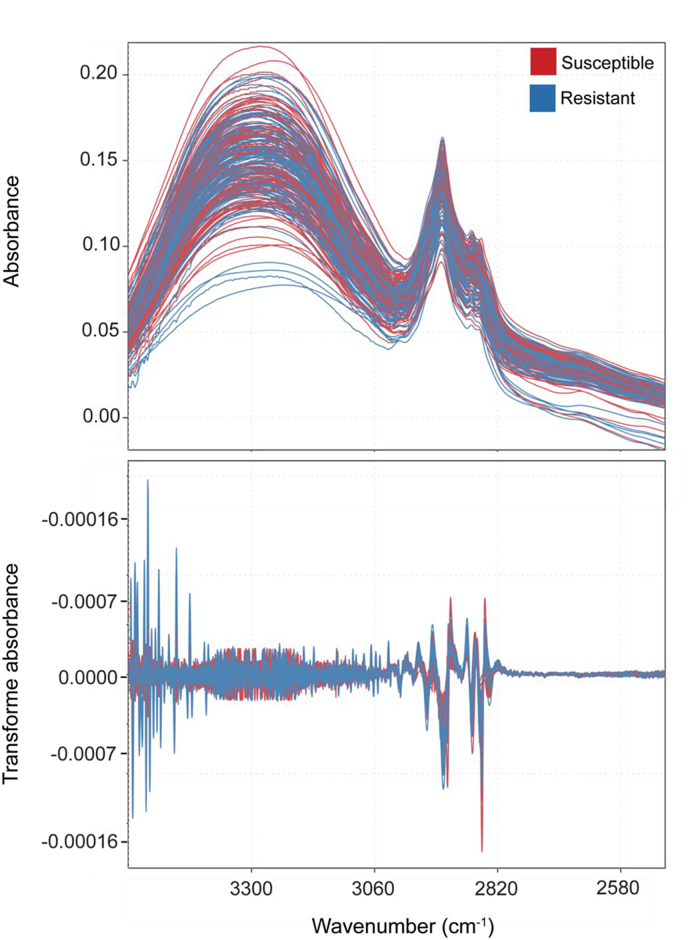
Raw and second-derivative transformed FT-IR spectra of extracted phenolics from phloem tissue of 5-year-old *Pinus taeda* seedlings representing families that are either susceptible or resistant to *Cronartium quercuum* f. sp. *fusiforme*, the causal agent of fusiform rust. Resistance was assigned based on breeding values across progeny tests.

After processing losses, 234 samples of phloem tissue from seven sites and three states were used in FT-IR analysis. The NMDS of all FT-IR spectra showed that there was strong grouping by site, but not by resistance class (Fig. S4). Similar to the NIR analyses, the PERMANOVA on the NMDS distance matrix indicated that the grouping of resistance class (R^2^ ​= ​0.637, p ​= ​0.167) and the interaction of resistance class and site (R^2^ ​= ​0.770, p ​= ​0.958) were not significant, but site alone was significant (R^2^ ​= ​0.008, p ​< ​0.001). Eight samples were identified as outliers by the chemometric pipeline and removed, resulting in a total of 226 samples. Results were similar to those of the NIR analysis, with the average raw and second-derivative transformed spectra not displaying noticeable differences between resistant and susceptible families (Fig. 4).

For the SVM approach, both the models built with either the 30 most and 30 least resistant trees or the 40 most and 40 least resistant trees performed similarly. The model built using the 30 most resistant and 30 most susceptible trees had 72.7 ​% training accuracy and 63.3 ​% testing accuracy (Table 6, Set 1). While in the model built using the 40 most resistant and 40 most susceptible trees, the training accuracy slightly decreased but the testing accuracy slightly increased. Using this data subset, the training accuracy was 70.1 ​% and the testing accuracy was 65.0 ​% (Table 6, Set 2). The model testing accuracy, however, drastically decreased to 54.3 ​% when including trees with intermediate breeding values, despite a high training accuracy of 83.6 ​% (Table 6, Set 3).

**Table 6:** Parameters and average accuracies of the three support vector machine (SVM) models with random forest-selected variables built with laboratory-based FT-IR spectra of extracted phenolics from phloem tissue and used to predict the resistance of 5-yr old loblolly *Pinus taeda* seedlings representing families that are either susceptible or resistant to *Cronartium quercuum* f. sp. *fusiforme*. Training and testing groups were portioned 70 ​% and 30 ​%, respectively, with equal resistance class representation. Model accuracies were averaged across 100 iterations of the data splitting. The best model is highlighted in bold.

Set	Data used	N	Group	Average accuracy ±SE
1	30 most resistant and 30 most susceptible trees	60	**Training**	0.727 ​± ​0.004
			**Testing**	0.633 ​± ​0.010
2	40 most resistant and 40 most susceptible trees	80	Training	**0.701 ​± ​0.004**
			Testing	**0.650 ​± ​0.009**
3	All breeding values	210	Training	0.836 ​± ​0.002
			Testing	0.543 ​± ​0.005

Using the sPLS-DA approach, overall predictive accuracy decreased for all data sets. Using the 30 most resistant and 30 most susceptible trees resistant trees, the training accuracy was 77.0 ​% and the testing accuracy was 47.7 ​% (Table 7, Set 1). When using the 40 most resistant and 40 most susceptible trees, the training accuracy was 78.6 ​% and the testing accuracy was 48.3 ​% (Table 7, Set 2). Finally, including trees with intermediate breeding values, the training accuracy was 71.9 ​% and the testing accuracy was 47.1 ​% (Table 7, Set 3). The sensitivity and specificity of the different model forms again followed a pattern similar to the one of the accuracies for both classification approaches (Table S3). Of the bands identified as important for classification, either by random forest or sPLS-DA, only four (2626, 2719, 2437, and 2491 ​cm^−1^) were overlapping across the different models (Table 3).

**Table 7:** Parameters and average accuracies of the sparse partial least squares discriminatory analysis (sPLS-DA) built with laboratory-based FT-IR spectra of extracted phenolics from phloem tissue and used to predict the resistance of 5-yr old *Pinus taeda* seedlings representing families that are either susceptible or resistant to *Cronartium quercuum* f. sp. *fusiforme*. Training and testing groups were portioned 70 ​% and 30 ​%, respectively, with equal resistance class representation. Model accuracies were averaged across 100 iterations of the data splitting. The best model is highlighted in bold.

Set	Data used	N	Group	Average accuracy ±SE
1	30 most resistant and 30 most susceptible trees	60	Training	0.770 ​± ​0.003
			Testing	0.477 ​± ​0.009
2	40 most resistant and 40 most susceptible trees	80	**Training**	**0.786** ​**±** ​**0.003**
			**Testing**	**0.483** ​**±** ​**0.008**
3	All breeding values	170	Training	0.719 ​± ​0.002
			Testing	0.471 ​± ​0.005

## 4 Discussion

The goal of this study was to determine if vibrational spectroscopy could be used to aid the phenotyping process for fusiform rust resistance in loblolly pine trees. We tested two different vibrational spectroscopy-based analytical approaches that showed promise in previous studies – the analysis of non-processed plant tissues with a handheld NIR device and the analysis of phenolic extracts with a benchtop FT-IR. Our hope was that the feasibility and efficiency of the handheld device could forgo the higher resolution of laboratory-based equipment, which requires sample collection, storage, and preparation. Our findings show that using vibrational spectroscopy, the resistance of loblolly pine trees could be correctly classified with up to 68.7 ​% accuracy. Moreover, models built with NIR spectra were comparable, and slightly better, than those built with FT-IR spectra and therefore validate the use of in-field, portable phenotyping.

Three different iterations of the data were used to test the model's capacity for resistance classification. Results from every model consistently showed that, as expected [32,42], the highest accuracies, sensitivities, and specificities occurred when using conservatively defined resistance classes (e.g., trees from the most resistant and susceptible families), and that adding more intermediate classes progressively lowered the performance. This implies that the methods are appropriate for classifying very resistant or susceptible trees, but not adequate to rank all genotypes. From a breeding operational standpoint, the ability to correctly classify extreme phenotypes is still very relevant and more important than correctly classifying intermediate phenotypes, which are not a target of breeding efforts anyway. The advantage for breeders of investing resources only in individuals that are likely very resistant, while discarding individuals that are likely very susceptible or intermediate, offsets the potential cost of including some intermediate phenotypes in the selection. Additionally, we tested the sensitivity (i.e., true positive rate) and specificity (i.e., true negative rate) to determine if there was bias for one phenotype over the other. As expected, both of these metrics were highest in the best performing, most conservative models for predictive accuracy. Specificity, however, was slightly higher than sensitivity, indicating a higher rate of correctly classifying susceptible individuals [58]. This can help with the issue of escaped susceptibles (i.e., susceptible individuals that are incorrectly classified as resistant), which are particularly detrimental to breeding efforts, especially when disease pressure is low, and likely offsets the potential missed opportunity cost of discarding highly resistant families.

To test the optimal model, we used two different supervised classification approaches: sPLS-DA and SVM with random forest. The sPLS-DA models identify variables that are conducive to predicting phenotype, similar to the random forest approach. In addition, the model automatically optimizes the number of variables and the number of components through cross-validation, which is helpful when using high dimensional datasets [59]. The SVM approach, on the other hand, does not have variable selection, which is why the random forest model was used. Both approaches yielded very similar results, which we interpreted as a confirmation of their robustness. Since variable selection is being used in a single framework with sPLS-DA rather than multiple decision trees (i.e., SVM with random forest), we suggest this former method for further investigations. To highlight spectral variables that may be associated with loblolly pine resistance mechanisms against fusiform rust, we reported the spectral bands that were more often selected by the algorithms and, specifically, those that overlapped across the different datasets and models. We found that the wavenumbers identified and subsequently used in the highest performing models were centralized in the first overtone region, which is associated with compounds such a lignin, cellulose, and sugars [60].

When comparing the classification performance parameters from both vibrational spectroscopy-based analytical approaches, the NIR one, surprisingly, outperformed the FT-IR one. The benchtop FT-IR, with which we analyzed phenolic extracts, collects high-resolution data from the mid-infrared region providing almost 7000 variables for each sample collected. The mid-infrared spectral range includes the ‘fingerprint region,’ which captures many sharp peaks that provide detailed information about a sample's physiochemical composition [61]. The NIR device, which we used it to directly analyze non-processed plant tissues, on the other hand, provides only 250 interpolated variables from the electromagnetic (i.e., near-infrared) region for each sample. The near-infrared region is less sensitive and with bands that are lower in intensity, which perhaps is why it is less established as a tool in the life sciences [62]. All told, given its high sensitivity, we expected spectra from the FT-IR approach to display starker differences between phenotypes as compared to those of the NIR approach. One reason for the difference in performance might be that the tools measure different regions of the infrared spectrum, which correspond to different functional groups: the NIR targets hydrogen-based functional groups [63], while the FT-IR targets organic compounds such as proteins, carotenoids, and polysaccharides [64]. Perhaps the hydrogen-based functional groups are more important in loblolly pine resistance mechanism against fusiform rust. Another reason could be the lack of an association between constitutive phenolics and resistance against fusiform rust, as also suggested by the fact that the spectral bands more often selected by the classifying models were centralized in the first overtone region. The actual mechanism of the induced defense response remains unclear [12] and might rely on more than one group of secondary metabolites. Since for the FT-IR analysis we mainly targeted phenolics based on their known association with defense against biotic attackers [65,66], we might have lost discriminating molecules during the extraction protocol. The portable NIR device, on the other hand, scans non-processed plant tissue, hence capturing the spectral signal of the totality of different molecules and structural make up present in the sample.

Phloem was also found to be a better indicator of fusiform rust resistance than needles. Indeed, leaf chemistry tends to be more variable and sensitive to site conditions such as moisture and soil nutrients [[67], [68], [69]]. These findings corroborate another study using FT-IR to phenotype for ash dieback resistance in ash trees, which showed that leaf tissue had a stronger association with site conditions, while phloem tissue was less influenced by the site and a stronger indicator of phenotype [42]. Moreover, while not fully confirmed, it is likely that loblolly pine resistance to fusiform rust lies in stem tissues [70].

One limitation of our best performing models was the limited sample sizes. In the most conservative models (built with the 30 most resistant and 30 most susceptible trees), there were 42 samples for the training set and 18 samples used to test the model. The use of limited sample sizes in machine learning is not uncommon but presents problems in overfitting and less statistical power for pattern recognition [71]. Moreover, limited sample size is a common problem in forestry as sample collection can be complicated by land ownership and management, the longevity of the species, biogeographical differences, and the size of the individual trees [72]. Regardless, our study shows that there is indeed potential in using vibrational spectroscopy tools for this pathosystem; though, a model built with more samples would be ideal before implementation.

There was also a strong association between the time of sampling at different locations and spectra from both devices. This is not unexpected given that loblolly pine in the southern United States undergoes significant phenological changes during the month of February: the trees just coming out of dormancy, are mobilizing water and nutrients, and are about to set seed [73]. Phenology has a strong effect on plant biochemistry [74], which was reflected in the results and may have introduced noise into the models, possibly explaining the lower performance when introducing more intermediate phenotypes. Our sampling time was limited to February due to logistic time constraints, but future studies in this system may want to consider the strong effect phenology has on spectral readings and favor sampling times in which fewer phenological changes are expected. The strong grouping can also be attributed to site effects, as factors such as biotic pressures, climate, and resource availability (which can change depending on the management objectives of a stand) are bound to be heterogenous and subsequently influence the chemical composition of a plant [75]. Moreover, NIR spectroscopic measurements can be significantly affected by the temperature of the samples, hence, different ambient temperatures on the different sampling days might have added another layer of variation [e.g., Ref. [76]. The introduction of site and sample condition heterogeneity, obtained by sampling at multiple sites, however, was by design, as we wanted to test if we could find underlying associations between the spectra of a tree and its resistance to fusiform rust that could be used regardless of site location or sampling conditions. As explained by Villari et al. [42], phenotyping models based on vibrational spectroscopy analytical approaches have to be able to detect a spectral signature regardless of the sites, otherwise, they would not be useful for breeding operations performed at a different location than the experimental one. The introduction of site heterogeneity, however, comes at the cost of introducing additional noise, which may have lowered the classification accuracies, especially in the models using more intermediate breeding values.

## 5 Conclusions

Pairing vibrational spectroscopy with multivariate statistics and machine learning provides an objective and sophisticated approach to classifying loblolly pine resistance against fusiform rust. Here, we show that a portable NIR is effective in providing measurements that can be delineated by commonly used classification methods (i.e., SVM, sPLS-DA). We developed models that classified trees as resistant or susceptible, even though all trees were asymptomatic. While the highest accuracy was only 69 ​%, this study provides a proof-of-concept for using in-field NIR spectroscopy for selection and breeding. The device itself is lightweight, requires only a small sample of tissue, and takes seconds to measure. Supplementing the current fusiform rust selection process with this method might give tree breeders an additional step of verification on an individual's phenotype, reducing the chance of misclassification and ‘escaped susceptibles,’ and adding an operational advantage to the process. This study can also be a framework for using this approach in the selection process of loblolly pine against other economically important diseases, such as pitch canker (caused by *Fusarium circinatum* Nirenberg & O'Donnell) and brown spot needle blight [caused by *Lecanosticta acicola* (von Thümen) Sydow]. This approach supplements the modern technology and sophisticated statistical analyses that provide important tools for the forestry sector to optimize forest health and management.

## CRediT statement

**Kitt G Payn**: Writing – review & editing, Resources, Funding acquisition. **Anna O Conrad**: Writing – review & editing, Methodology. **Kamal JK Gandhi**: Writing – review & editing, Funding acquisition. **Caterina Villari**: Writing – review & editing, Supervision, Funding acquisition, Methodology, Conceptualization. **Cristián R Montes**: Writing – review & editing, Supervision, Methodology, Conceptualization. **Trevor D Walker**: Writing – review & editing, Resources, Funding acquisition. **Simone Lim-Hing**: Writing – original draft, Visualization, Software, Methodology, Investigation, Formal analysis.

## Data availability

The data collected and used in this study contains proprietary information of the Southern Pine Health Research Cooperative, University of Georgia and cannot be shared. The code used for analysis is available from the corresponding author, S.L., upon reasonable request.

## Funding

This research was funded by the United States Forest Service, Forest Health Protection Special Technology Development Program (grant number 20-DG-11083150-003) and the Southern Pine Health Research Cooperative (SPHRC) at the University of Georgia (Athens, Georgia, United States). We would like to thank the Cooperative Tree Improvement at NC State University (Raleigh, North Carolina, United States) for providing data, which was made possible because of the establishment, management, and measurement of tests by members of the Cooperative. Funding for the Cooperative was also provided by the Department of Forestry and Environmental Resources in the College of Natural Resources at North Carolina State University and by USDA National Institute of Food and Agriculture McIntire-Stennis Project NCZ04149. We would also like to thank ArborGen, Georgia Forestry Commission, IFCO, Rayonier, Westervelt, and Weyerhaeuser for providing access to their sites and allowing us to sample. We thank the three anonymous reviewers for their helpful comments that improved the paper's quality.

## Declaration of competing interest

The authors declare that they have no known competing financial interests or personal relationships that could have appeared to influence the work reported in this paper.

## References

[1] Guo J., Prestemon J., Johnston C. (2023). Forest market outlook in the Southern United States. For. Pol. Econ..

[2] Prestemon J.P., Abt R.C. (2002). Southern forest resource assessment highlights: the southern timber market to 2040. J. For..

[3] Powers H.R., Schmidt R.A., Snow G.A. (1981). Current status and management of fusiform rust on southern pines. Annu. Rev. Phytopathol..

[4] Czabator F.J. (1971).

[5] Schmidt R.A. (2003). Fusiform rust of southern pines: a major success for forest disease management. Phytopathology.

[6] Cubbage F.W., Pye J.M., Holmes T.P., Wagner J.E. (2000). An economic evaluation of fusiform rust protection research. South. J. Appl. For..

[7] Kuhlman E.G., Powers H.J. (1988). Resistance responses in half-sib loblolly pine progenies after inoculation with *Cronartium quercuum* f. sp. *fusiforme*. Phytopathology.

[8] Susaeta A. (2020). Implications of future risk of fusiform rust on optimal forest management of even-aged slash pine plantations. For. Pol. Econ..

[9] Carey W.A., Kelley W.D. (1993). Seedling production trends and fusiform rust control practices at southern nurseries, 1981-1991. South. J. Appl. For..

[10] Lim-Hing S., Montes C.R., Walker T.D., Shalizi M.N., Gandhi K.J., Villari C. (2024). Enhanced fusiform rust hazard maps for loblolly pine: incorporating genotype and climate to predict disease. For. Ecol. Manag..

[11] McKeand S., Mullin T., Byram T., White T. (2003). Deployment of genetically improved loblolly and slash pines in the south. J. For..

[12] Sniezko R.A., Smith J., Liu J.-J., Hamelin R.C. (2014). Genetic resistance to fusiform rust in southern pines and white pine blister rust in white pines—a contrasting tale of two rust pathosystems—current status and future prospects. Forests.

[13] McKeand S.E., Li B., Amerson H.V. (1999). Genetic variation in fusiform rust resistance in loblolly pine across a wide geographic range. Silvae Genet..

[14] Niks R.E., Qi X., Marcel T.C. (2015). Quantitative resistance to biotrophic filamentous plant pathogens: concepts, misconceptions, and mechanisms. Annu. Rev. Phytopathol..

[15] Wilcox P.L., Amerson H.V., Kuhlman E.G., Liu B.-H., O'Malley D.M., Sederoff R.R. (1996). Detection of a major gene for resistance to fusiform rust disease in loblolly pine by genomic mapping. Proc. Natl. Acad. Sci..

[16] Kubisiak T.L., Anderson C.L., Amerson H.V., Smith J.A., Davis J.M., Nelson C.D. (2011). A genomic map enriched for markers linked to Avr1 in *Cronartium quercuum* f.sp. *fusiforme*. Fungal Genet. Biol..

[17] McKeand S.E., Frampton Jr LJ. (1984).

[18] Quesada T., Resende Jr MF., Muñoz P., Wegrzyn J.L., Neale D.B., Kirst M. (2014). Mapping fusiform rust resistance genes within a complex mating design of loblolly pine. Forests.

[19] Kubisiak T.L., Amerson H.V., Nelson C.D. (2005). Genetic interaction of the fusiform rust fungus with resistance gene Fr 1 in loblolly pine. Phytopathology.

[20] Remington D.L., O'Malley D.M. (2000). Evaluation of major genetic loci contributing to inbreeding depression for survival and early growth in a selfed family of *Pinus taeda*. Evolution.

[21] Kuhlman E.G. (1992). Interaction of virulent single-gall rust isolates of *Cronartium quercuum* f. sp. *fusiforme* and resistant families of loblolly pine. For. Sci..

[22] Bock C.H., Poole G.H., Parker P.E., Gottwald T.R. (2010). Plant disease severity estimated visually, by digital photography and image analysis, and by hyperspectral imaging. Crit. Rev. Plant Sci..

[23] Singh A., Jones S., Ganapathysubramanian B., Sarkar S., Mueller D., Sandhu K. (2021). Challenges and opportunities in machine-augmented plant stress phenotyping. Trends Plant Sci..

[24] (2023). Annual Report of the Cooperative Tree Improvement Program.

[25] Walker T.D., McKeand S.E. (2018). Fusiform rust hazard mapping for loblolly pine in the southeastern United States using progeny test data. J. For..

[26] Kayihan G.C., Huber D.A., Morse A.M., White T.L., Davis J.M. (2005). Genetic dissection of fusiform rust and pitch canker disease traits in loblolly pine. Theor. Appl. Genet..

[27] Cozzolino D. (2014). Use of infrared spectroscopy for in-field measurement and phenotyping of plant properties: instrumentation, data analysis, and examples. Appl. Spectrosc. Rev..

[28] Moros J., Garrigues S., Guardia M de la (2010). Vibrational spectroscopy provides a green tool for multi-component analysis. Trends Anal. Chem..

[29] Li-Chan E., Chalmers J.M., Griffiths P.R. (2010).

[30] Kendall C., Isabelle M., Bazant-Hegemark F., Hutchings J., Orr L., Babrah J. (2009). Vibrational spectroscopy: a clinical tool for cancer diagnostics. Analyst.

[31] Efeoglu E., Maher M.A., Casey A., Byrne H.J. (2018). Toxicological assessment of nanomaterials: the role of in vitro Raman microspectroscopic analysis. Anal. Bioanal. Chem..

[32] Conrad A.O., Bonello P. (2016). Application of infrared and Raman spectroscopy for the identification of disease resistant trees. Front. Plant Sci..

[33] Danzi D., De Paola D., Petrozza A., Summerer S., Cellini F., Pignone D. (2022). The use of near-infrared imaging (NIR) as a fast non-destructive screening tool to identify drought-tolerant wheat genotypes. Agriculture.

[34] Madurapperumage A., Johnson N., Thavarajah P., Tang L., Thavarajah D. (2022). Fourier-transform infrared spectroscopy (FTIR) as a high-throughput phenotyping tool for quantifying protein quality in pulse crops. Plant Phenome J..

[35] Monti F., Dell'Anna R., Sanson A., Fasoli M., Pezzotti M., Zenoni S. (2013). A multivariate statistical analysis approach to highlight molecular processes in plant cell walls through ATR FT-IR microspectroscopy: the role of the α-expansin PhEXPA1 in *Petunia hybrida*. Vib. Spectrosc..

[36] Conrad A.O., Li W., Lee D.-Y., Wang G.-L., Rodriguez-Saona L., Bonello P. (2020). Machine learning-based presymptomatic detection of rice sheath blight using spectral profiles. Plant Phenomics.

[37] Sylvain T., Cecile L.-G. (2018). Disease identification: a review of vibrational spectroscopy applications. Compr. Anal. Chem..

[38] Mukrimin M., Conrad A.O., Kovalchuk A., Julkunen-Tiitto R., Bonello P., Asiegbu F.O. (2019). Fourier-transform infrared (FT-IR) spectroscopy analysis discriminates asymptomatic and symptomatic Norway spruce trees. Plant Sci..

[39] Fearer C.J., Conrad A.O., Marra R.E., Georskey C., Villari C., Slot J. (2022). A combined approach for early in-field detection of beech leaf disease using near-infrared spectroscopy and machine learning. Frontiers. For. Glob. Change.

[40] Conrad A.O., Rodriguez-Saona L.E., McPherson B.A., Wood D.L., Bonello P. (2014). Identification of *Quercus agrifolia* (coast live oak) resistant to the invasive pathogen *Phytophthora ramorum* in native stands using Fourier-transform infrared (FT-IR) spectroscopy. Front. Plant Sci..

[41] Conrad A.O., Villari C., Sherwood P., Bonello P.E. (2020). Phenotyping Austrian pine for resistance using Fourier-transform infrared spectroscopy. Arboric. Urban For..

[42] Villari C., Dowkiw A., Enderle R., Ghasemkhani M., Kirisits T., Kjær E.D. (2018). Advanced spectroscopy-based phenotyping offers a potential solution to the ash dieback epidemic. Sci. Rep..

[43] Isik F., McKeand S.E. (2019). Fourth cycle breeding and testing strategy for *Pinus taeda* in the NC state university cooperative tree improvement Program. Tree Genet. Genomes.

[44] Gilmour A.R., Gogel B.J., Cullis B.R., Welham S.J., Thompson R., Butler D. (2014).

[45] Isik F., Holland J., Maltecca C. (2017).

[46] Lattanzio V., Lattanzio V.M., Cardinali A. (2006). Role of phenolics in the resistance mechanisms of plants against fungal pathogens and insects. Phytochemistry: Adv. Res..

[47] López-Goldar X., Villari C., Bonello P., Borg-Karlson A.K., Grivet D., Zas R. (2018). Inducibility of plant secondary metabolites in the stem predicts genetic variation in resistance against a key insect herbivore in maritime pine. Front. Plant Sci..

[48] R Core Team (2022).

[49] Oksanen J., Kindt R., Legendre P., O'Hara B., Stevens M.H.H., Oksanen M.J. (2007). The vegan package. Community Ecology Package.

[50] Bande M.F., de la Fuente M.O., Galeano P., Nieto A., Garcia-Portugues E., de la Fuente M.M.O. (2022).

[51] Ramsay J.O., Wickham H., Ramsay M.J., deSolve S. (2022).

[52] Kucheryavskiy S. (2020). mdatools–R package for chemometrics. Chemometr. Intell. Lab. Syst..

[53] Meyer D., Dimitriadou E., Hornik K., Weingessel A., Leisch F., Chang C.-C. (2019). Package ‘e1071.’. The R Journal.

[54] Genuer R., Poggi J.-M., Tuleau-Malot C. (2015). VSURF: an R package for variable selection using random forests. The R Journal.

[55] Rohart F., Gautier B., Singh A., Lê Cao K.-A. (2017). mixOmics: an R package for ‘omics feature selection and multiple data integration. PLoS Comput. Biol..

[56] Krysa M., Szymańska-Chargot M., Zdunek A. (2022). FT-IR and FT-Raman fingerprints of flavonoids – a review. Food Chem..

[57] Kuhn M. (2012). http://Https://Cran.r-Project.Org/Package=Caret.

[58] Altman D.G., Bland J.M. (1994). Diagnostic tests. 1: sensitivity and specificity. Br. Med. J..

[59] Lê Cao K.-A., Boitard S., Besse P. (2011). Sparse PLS discriminant analysis: biologically relevant feature selection and graphical displays for multiclass problems. BMC Bioinf..

[60] Curran P.J. (1989). Remote sensing of foliar chemistry. Rem. Sens. Environ..

[61] Ellis D.I., Goodacre R. (2006). Metabolic fingerprinting in disease diagnosis: biomedical applications of infrared and Raman spectroscopy. Analyst.

[62] Beć K.B., Grabska J., Huck C.W. (2020). Near-Infrared spectroscopy in bio-Applications. Molecules (Basel).

[63] Siesler H.W., Kawata S., Heise H.M., Ozaki Y. (2008).

[64] Rodriguez-Saona L.E., Allendorf M.E. (2011). Use of FTIR for rapid authentication and detection of adulteration of food. Annu. Rev. Food Sci. Technol..

[65] Franceschi V.R., Krokene P., Christiansen E., Krekling T. (2005). Anatomical and chemical defenses of conifer bark against bark beetles and other pests. New Phytol..

[66] Sherwood P., Bonello P. (2013). Austrian pine phenolics are likely contributors to systemic induced resistance against *Diplodia pinea*. Tree Physiol..

[67] Anderson J.V., Chevone B.I., Hess J.L. (1992). Seasonal variation in the antioxidant system of eastern white pine needles 1: evidence for thermal dependence. Plant Physiol..

[68] Cunningham S.A., Summerhayes B., Westoby M. (1999). Evolutionary divergences in leaf structure and chemistry, comparing rainfall and soil nutrient gradients. Ecol. Monogr..

[69] López-Orenes A., Bueso M.C., Conesa H., Calderón A.A., Ferrer M.A. (2018). Seasonal ionomic and metabolic changes in Aleppo pines growing on mine tailings under Mediterranean semi-arid climate. Sci. Total Environ..

[70] Jewell F.F., Speirs D.C. (1976). Histopathology of one-and two-year-old resisted infections by *Cronartium fusiforme* in slash pine. Phytopathology.

[71] Raudys S.J., Jain A.K. (1991). Small sample size effects in statistical pattern recognition: recommendations for practitioners. IEEE *Transactions on Pattern Analysis and Machine Intelligence*.

[72] Roman L.A., McPherson E.G., Scharenbroch B.C., Bartens J. (2013). Identifying common practices and challenges for local urban tree monitoring programs across the United States. Arboric. Urban For..

[73] Dougherty P.M., Whitehead D., Vose J.M. (1994). Environmental influences on the phenology of pine. Ecol. Bull..

[74] Jarzomski C.M., Stamp N.E., Bowers M.D. (2000). Effects of plant phenology, nutrients and herbivory on growth and defensive chemistry of plantain, *Plantago lanceolata*. Oikos.

[75] Moore B.D., Andrew R.L., Külheim C., Foley W.J. (2014). Explaining intraspecific diversity in plant secondary metabolites in an ecological context. New Phytol..

[76] Cozzolino D., Liu L., Cynkar W.U., Dambergs R.G., Janik L., Colby C.B., Gishen M. (2007). Effect of temperature variation on the visible and near infrared spectra of wine and the consequences on the partial least square calibrations developed to measure chemical composition. Anal. Chim. Acta.

