# Supplementary material for: Near-infrared spectroscopy as a high-throughput phenotyping method for fusiform rust resistance in loblolly pine
Source: Plant Phenomics. 2025 Jun 6;7(3):100066. doi: 10.1016/j.plaphe.2025.100066 (PMC12710050; doi:10.1016/j.plaphe.2025.100066)
Supplement: Multimedia component 1 [file mmc1.docx]

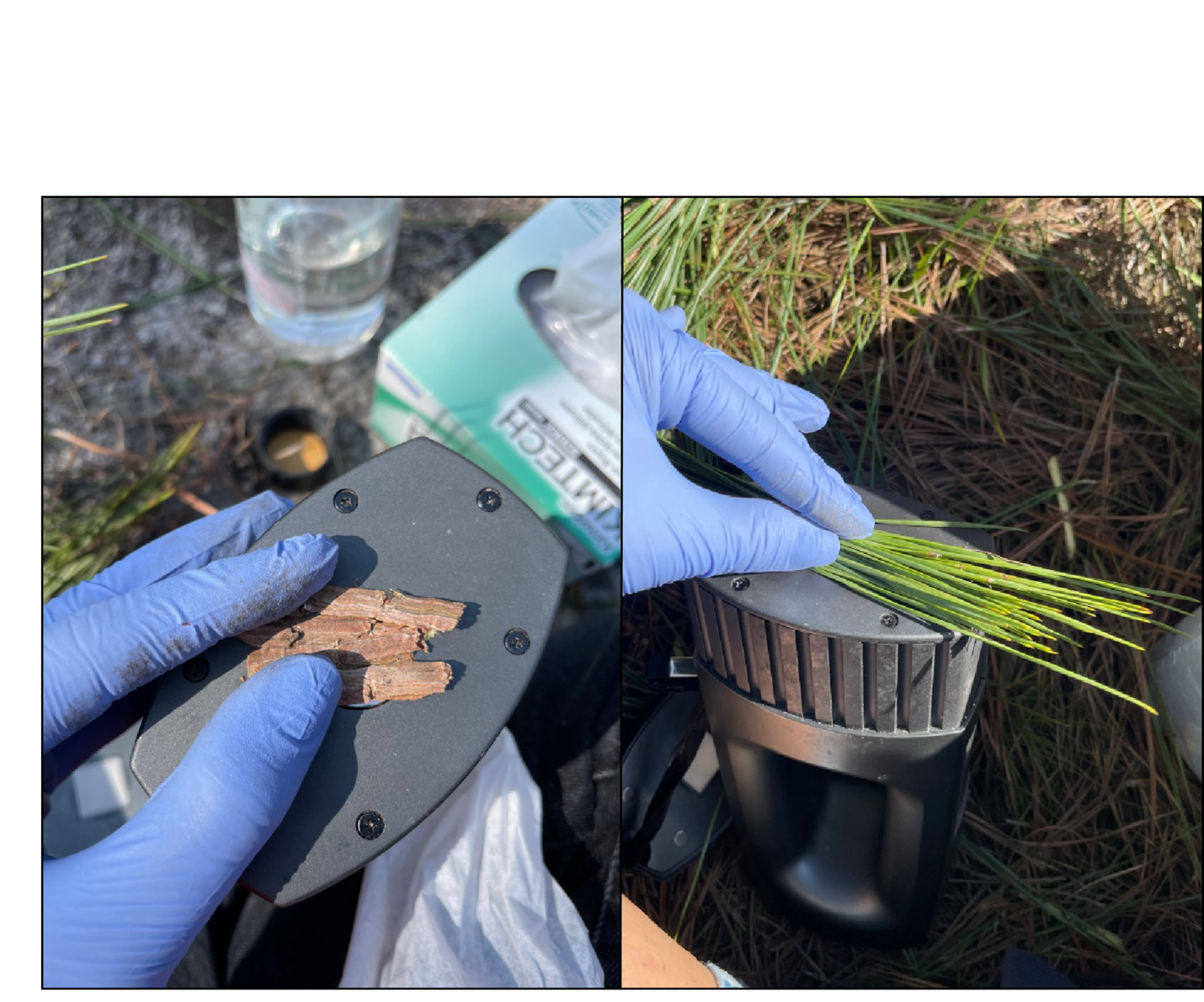


Fig. S1. Photos of NIR spectra acquisition for phloem (left) and needles (right) from 5-year-old loblolly pine (*Pinus taeda*) seedlings. For FT-IR analysis, the same tissue from another side of the branch was collected and stored in dry ice until transported to the lab for phenolic extraction.


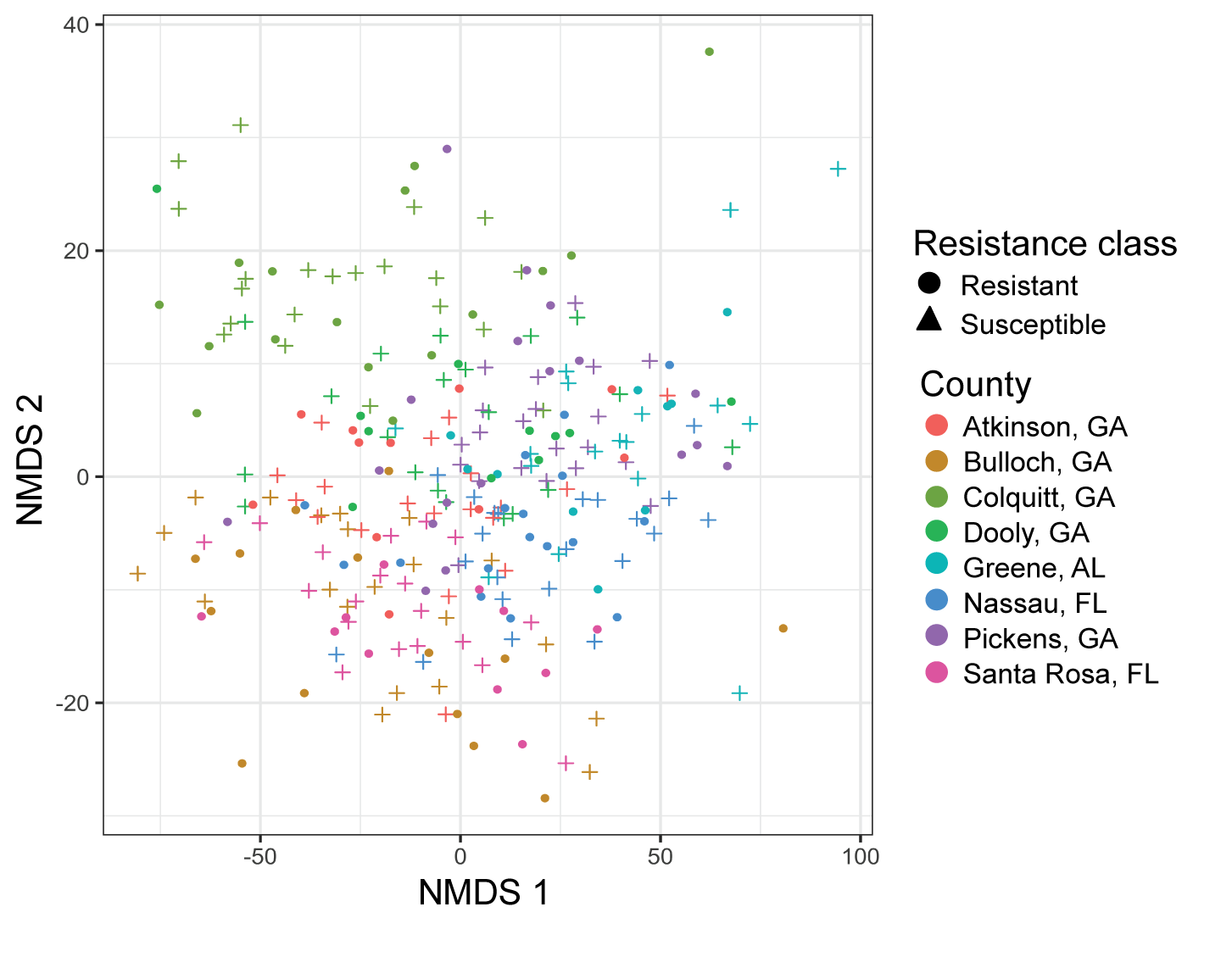


Fig. S2. Non-parametric multidimensional scaling of NIR spectra from phloem tissue of loblolly pine (*Pinus taeda*) trees representing families either resistant or susceptible to fusiform rust (caused by *Cronartium quercuum* f. sp. *fusiforme*). Shape indicates resistance class and color indicates site location. Ordination stress for the NMDS was 0.019. A PERMANOVA on the NMDS distance matrix indicated that grouping resistance class (R^2^= 0.008, p = 0.613) and the interaction of resistance class and site (R^2^ = 0.016, p = 0.422) were not significant, but site alone was (R^2^ = 0.381, p < 0.001).


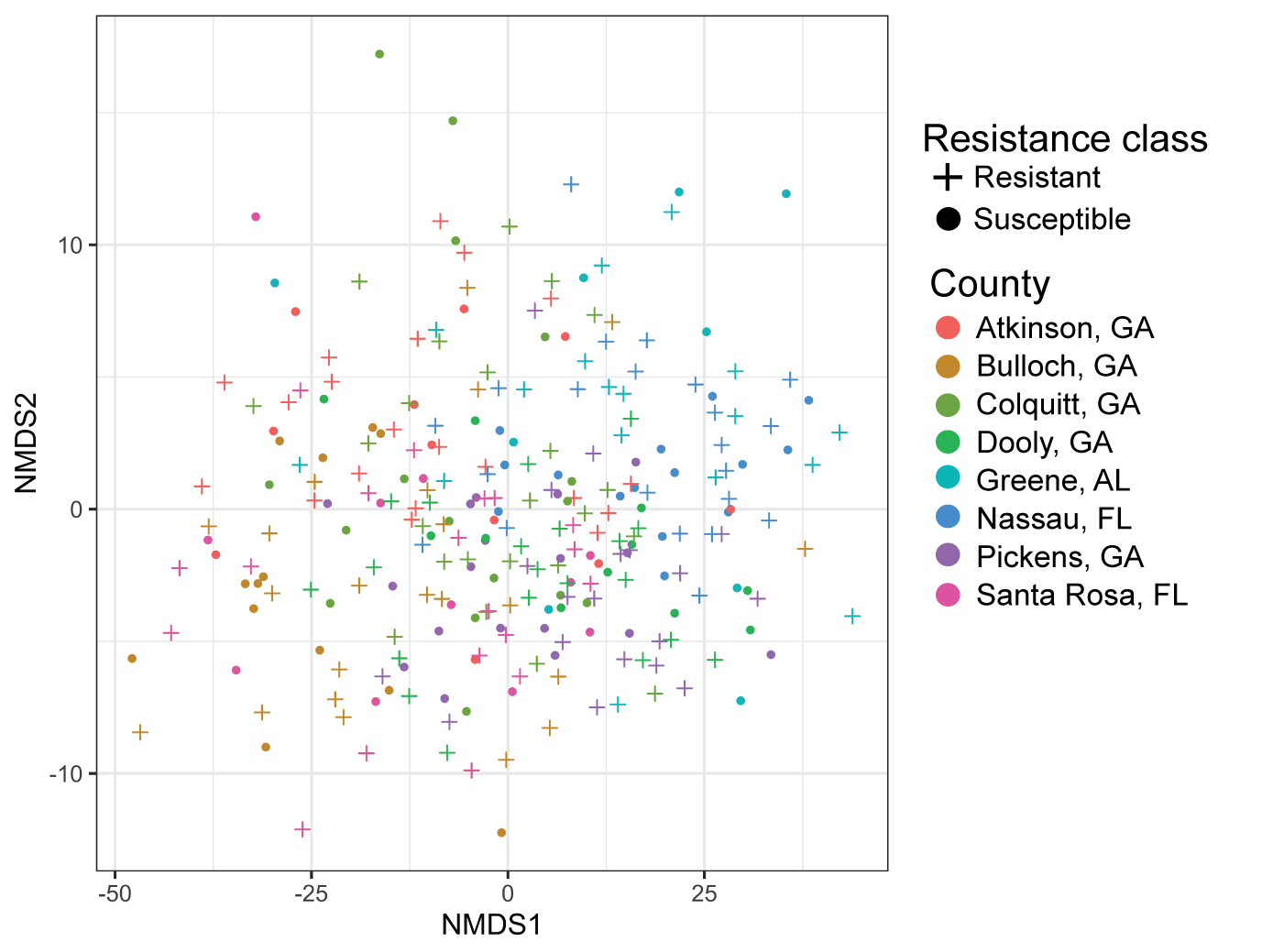


Fig. S3. Non-parametric multidimensional scaling of NIR spectra from needle tissue of loblolly pine (*Pinus taeda*) trees representing families either resistant or susceptible to fusiform rust (caused by *Cronartium quercuum* f. sp. *fusiforme*). Shape indicates resistance class and color indicates site location. Ordination stress for the NMDS was 0.037. A PERMANOVA on the NMDS distance matrix indicated that grouping resistance class (R^2^= 0.004, p = 0.194) and the interaction of resistance class and site (R^2^ = 0.024, p = 0.168) were not significant, but site alone was (R^2^ = 0.373, p < 0.001).


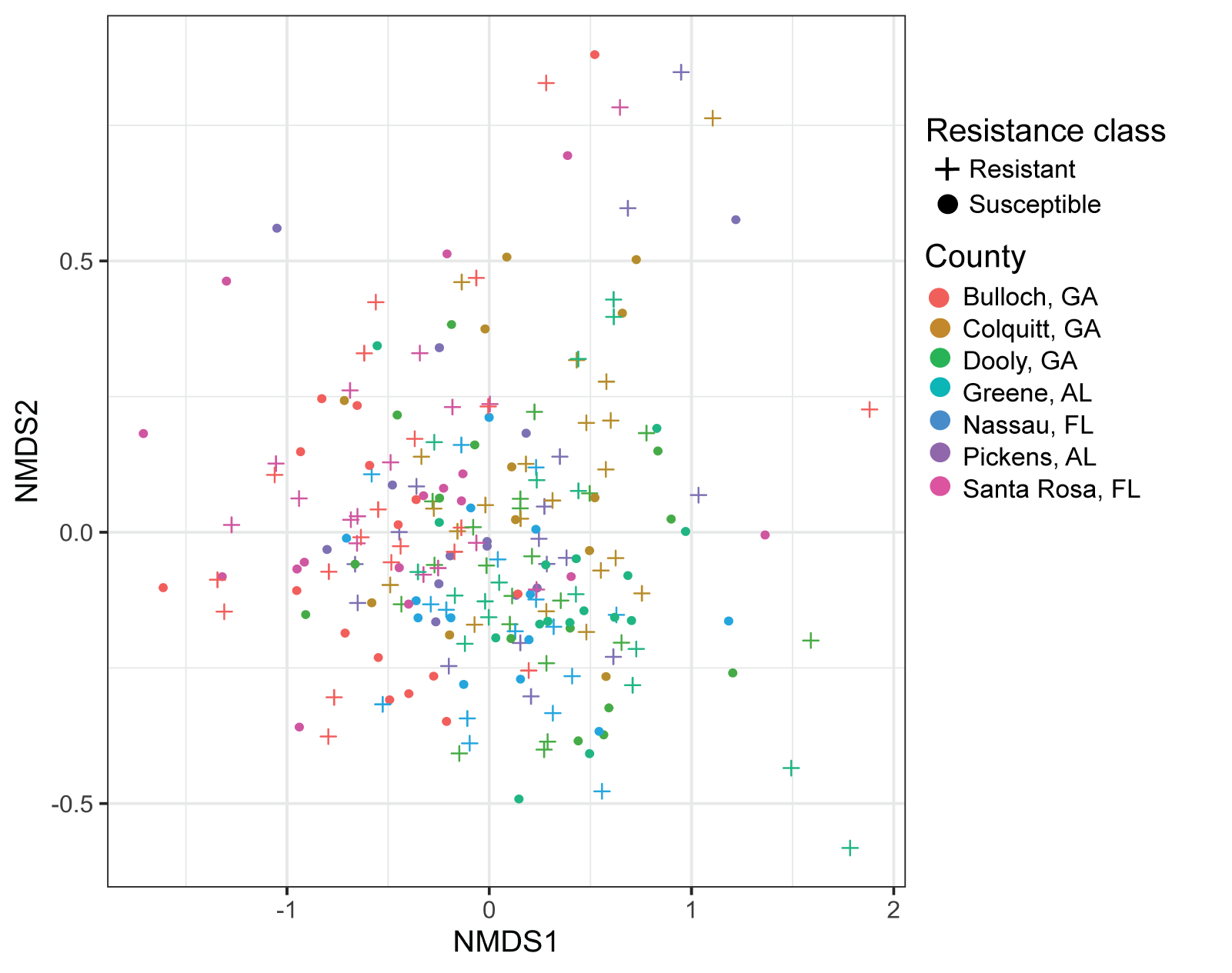


Fig. S4. Non-parametric multidimensional scaling of FT-IR spectra of extracted phenolics from phloem tissue of loblolly pine (*Pinus taeda*) trees representing families either resistant or susceptible to fusiform rust (caused by *Cronartium quercuum* f. sp. *fusiforme*). Shape indicates resistance class and color indicates site location. Ordination stress for the NMDS was 0.130. A PERMANOVA on the NMDS distance matrix indicated that grouping resistance class (R^2^= 0.637, p = 0.167) and the interaction of resistance class and site (R^2^ = 0.770, p = 0.958) were not significant, but site alone was (R^2^ = 0.008, p < 0.001).

Table S1. Detailed information on the location and number of trees per each *Pinus taeda* family sampled in the study and used in the analysis. Family names are codified to protect proprietary information. Resistance was assigned based on breeding values across progeny tests.

|  |  | Site |  |  |  |  |  |  |  |
| --- | --- | --- | --- | --- | --- | --- | --- | --- | --- |
| Family | Resistance class | Atkinson | Bulloch | Colquitt | Dooly | Greene | Nassau | Pickens | Santa Rosa |
| X1 | Resistant | 2 | 0 | 0 | 2 | 2 | 0 | 0 | 2 |
| X2 | Susceptible | 2 | 1 | 1 | 1 | 0 | 2 | 2 | 2 |
| X3 | Susceptible | 0 | 2 | 2 | 0 | 0 | 2 | 2 | 0 |
| X4 | Resistant | 0 | 0 | 2 | 0 | 0 | 2 | 2 | 0 |
| X5 | Resistant | 0 | 1 | 2 | 0 | 0 | 2 | 2 | 0 |
| X6 | Resistant | 0 | 2 | 2 | 0 | 0 | 2 | 1 | 0 |
| X7 | Resistant | 0 | 2 | 2 | 0 | 0 | 2 | 2 | 0 |
| X8 | Susceptible | 0 | 1 | 1 | 0 | 0 | 1 | 1 | 0 |
| X9 | Susceptible | 1 | 0 | 0 | 2 | 2 | 0 | 0 | 1 |
| X10 | Susceptible | 0 | 2 | 2 | 0 | 0 | 2 | 2 | 0 |
| X11 | Resistant | 2 | 1 | 1 | 2 | 1 | 1 | 1 | 2 |
| X12 | Resistant | 2 | 0 | 0 | 2 | 2 | 0 | 0 | 2 |
| X13 | Susceptible | 0 | 1 | 2 | 0 | 0 | 2 | 2 | 0 |
| X14 | Resistant | 2 | 0 | 0 | 2 | 2 | 0 | 0 | 2 |
| X15 | Susceptible | 0 | 0 | 2 | 0 | 0 | 2 | 2 | 0 |
| X16 | Susceptible | 2 | 0 | 0 | 2 | 2 | 0 | 0 | 2 |
| X17 | Resistant | 0 | 2 | 2 | 0 | 0 | 2 | 1 | 0 |
| X18 | Susceptible | 2 | 2 | 2 | 2 | 2 | 0 | 2 | 2 |
| X19 | Susceptible | 0 | 2 | 2 | 0 | 0 | 2 | 2 | 0 |
| X20 | Resistant | 2 | 0 | 0 | 2 | 2 | 0 | 0 | 2 |
| X21 | Resistant | 0 | 2 | 1 | 0 | 0 | 2 | 2 | 0 |
| X22 | Resistant | 0 | 1 | 2 | 0 | 0 | 2 | 2 | 0 |
| X23 | Susceptible | 2 | 0 | 0 | 2 | 2 | 0 | 0 | 2 |
| X24 | Resistant | 0 | 2 | 2 | 0 | 0 | 2 | 2 | 0 |
| X25 | Susceptible | 0 | 1 | 0 | 0 | 0 | 2 | 2 | 0 |
| X26 | Resistant | 0 | 2 | 2 | 0 | 0 | 0 | 0 | 0 |
| X27 | Susceptible | 2 | 0 | 0 | 2 | 2 | 0 | 0 | 2 |
| X28 | Susceptible | 0 | 2 | 2 | 0 | 0 | 2 | 1 | 0 |
| X29 | Resistant | 0 | 2 | 2 | 0 | 0 | 2 | 2 | 0 |
| X30 | Resistant | 2 | 0 | 0 | 2 | 2 | 0 | 0 | 2 |
| X31 | Resistant | 1 | 2 | 2 | 2 | 2 | 2 | 2 | 2 |
| X32 | Resistant | 2 | 0 | 0 | 2 | 2 | 0 | 0 | 2 |
| X33 | Resistant | 2 | 0 | 0 | 2 | 1 | 0 | 0 | 2 |
| X34 | Resistant | 2 | 2 | 2 | 2 | 2 | 2 | 2 | 2 |
|  | Total trees | 30 | 35 | 40 | 31 | 28 | 40 | 39 | 31 |

Table S2. Average sensitivity (in this case, rate of correctly classifying resistance) and specificity (in this case, rate of correctly classifying susceptibility) of models that were built with field-based NIR spectra and used to predict the resistance of 5-yr old *Pinus taeda* seedlings representing families that are either susceptible or resistant to *Cronartium quercuum* f. sp. *fusiforme*.

|  | Model used |  |  |  |
| --- | --- | --- | --- | --- |
| **Tissue** |  | **Data used** | **Sensitivity** | **Specificity** |
| Phloem | Random forest and support vector machine | 30 most resistant and 30 most susceptible trees | 0.644 ± 0.017 | 0.718 ± 0.015 |
|  |  | 40 most resistant and 40 most susceptible trees | 0.680 ± 0.014 | 0.630 ± 0.018 |
|  |  | All breeding values | 0.509 ± 0.010 | 0.540 ± 0.009 |
|  | sPLS-DA | 30 most resistant and 30 most susceptible trees | 0.640 ± 0.016 | 0.672 ± 0.013 |
|  |  | 40 most resistant and 40 most susceptible trees | 0.517 ± 0.014 | 0.672 ± 0.013 |
|  |  | All breeding values | 0.521 ± 0.009 | 0.443 ± 0.009 |
| Needles | Random forest and support vector machine | 30 most resistant and 30 most susceptible trees | 0.600 ± 0.016 | 0.638 ± 0.016 |
|  |  | 40 most resistant and 40 most susceptible trees | 0.542 ± 0.015 | 0.638 ± 0.015 |
|  |  | All breeding values | 0.494 ± 0.010 | 0.475 ± 0.010 |
|  | sPLS-DA | 30 most resistant and 30 most susceptible trees | 0.479 ± 0.009 | 0.465 ± 0.010 |
|  |  | 40 most resistant and 40 most susceptible trees | 0.458 ± 0.015 | 0.449 ± 0.014 |
|  |  | All breeding values | 0.472 ± 0.006 | 0.470 ± 0.006 |

Table S3. Average sensitivity (in this case, rate of correctly classifying resistance) and specificity (in this case, rate of correctly classifying susceptibility) of models that were built with laboratory-based FT-IR spectra and used to predict the resistance of 5-yr old *Pinus taeda* seedlings representing families that are either susceptible or resistant to *Cronartium quercuum* f. sp. *fusiforme*.

| Model used |  |  |  |  |
| --- | --- | --- | --- | --- |
|  | **Data used** | **Average sensitivity ± SE** | **Average specificity ± SE** | |
| Random forest and support vector machine | 30 most resistant and 30 most susceptible trees | 0.589 ± 0.016 | 0.651 ± 0.016 |  |
|  | 40 most resistant and 40 most susceptible trees | 0.603 ± 0.014 | 0.647 ± 0.016 |  |
|  | All breeding values | 0.518 ± 0.009 | 0.383 ± 0.012 |  |
| sPLS-DA | 30 most resistant and 30 most susceptible trees | 0.470 ± 0.017 | 0.469 ± 0.019 |  |
|  | 40 most resistant and 40 most susceptible trees | 0.455 ± 0.016 | 0.497 ± 0.014 |  |
|  | All breeding values | 0.452 ± 0.009 | 0.466 ± 0.009 |  |
